# Thymoquinone Is a Multitarget Single Epidrug That Inhibits the UHRF1 Protein Complex

**DOI:** 10.3390/genes12050622

**Published:** 2021-04-22

**Authors:** Omeima Abdullah, Ziad Omran, Salman Hosawi, Ali Hamiche, Christian Bronner, Mahmoud Alhosin

**Affiliations:** 1 College of Pharmacy, Umm Al-Qura University, Makkah 21955, Saudi Arabia; oaabdullah@uqu.edu.sa (O.A.); ziadomran@hotmail.com (Z.O.); 2 Department of Biochemistry, Faculty of Science, Cancer and Mutagenesis Unit, King Fahd Medical Research Center, King Abdulaziz University, Jeddah 21589, Saudi Arabia; shosawi@kau.edu.sa; 3 Institut de Génétique et de Biologie Moléculaire et Cellulaire, CNRS UMR7104, INSERM U964, Université de Strasbourg, 67404 Illkirch, France; hamiche@igbmc.fr (A.H.); bronnerc@igbmc.fr (C.B.)

**Keywords:** thymoquinone, UHRF1, epigenetic drug, cancer, tumor suppressor gene

## Abstract

Silencing of tumor suppressor genes (TSGs) through epigenetic mechanisms, mainly via abnormal promoter DNA methylation, is considered a main mechanism of tumorigenesis. The abnormal DNA methylation profiles are transmitted from the cancer mother cell to the daughter cells through the involvement of a macromolecular complex in which the ubiquitin-like containing plant homeodomain (PHD), and an interesting new gene (RING) finger domains 1 (UHRF1), play the role of conductor. Indeed, UHRF1 interacts with epigenetic writers, such as DNA methyltransferase 1 (DNMT1), histone methyltransferase G9a, erasers like histone deacetylase 1 (HDAC1), and functions as a hub protein. Thus, targeting UHRF1 and/or its partners is a promising strategy for epigenetic cancer therapy. The natural compound thymoquinone (TQ) exhibits anticancer activities by targeting several cellular signaling pathways, including those involving UHRF1. In this review, we highlight TQ as a potential multitarget single epidrug that functions by targeting the UHRF1/DNMT1/HDAC1/G9a complex. We also speculate on the possibility that TQ might specifically target UHRF1, with subsequent regulatory effects on other partners.

## 1. Introduction

**Figure 1:**
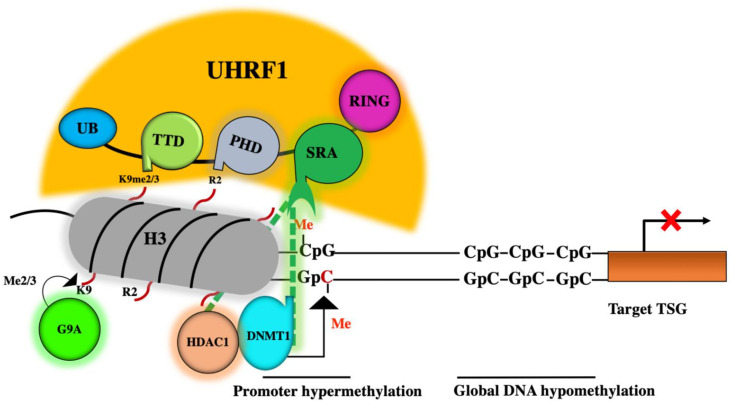
Role of the epigenetic reader UHRF1 (ubiquitin-like containing plant homeodomain (PHD) and interesting new gene (RING) finger domains 1) in epigenetic silencing of tumor suppressor genes (TSGs). During DNA replication, the SET and RING-associated (SRA) domain of UHRF1 can read methylated CpG sites (hemimethylated DNA) located with TSG promoter. Via the SRA domain, UHRF1 also recruits DNA methyltransferase 1 (DNMT1) and guides it to methylate the unmethylated cytosine of the newly synthetized DNA strand, leading to hypermethylation of the TSG promoter with a global hypomethylation. Through the plant homeodomain (PHD) domain, UHRF1 can bind to unmodified arginine 2 of histone 3 and via its tandem Tudor domain (TTD) domain, UHRF1 can recognize and bind to di or trimethylation of lysine 9 of histone 3 (H3K9me2 or H3K9me3). UHRF1 also uses its SRA domain to recruit histone deacetylase 1 (HDAC1) and recruits histone methyltransferase G9a, leading to histone 3 deacetylation and methylation, respectively. The consequence is the epigenetic silencing of TSGs.

Epigenetic silencing of tumor suppressor genes (TSGs) is considered a main mechanism driving cancer initiation and progression [1,2,3]. Consequently, the reversibility of epigenetic changes has attracted attention and highlighted these changes as interesting targets in the prevention and treatment of cancer. The main epigenetic mechanisms controlling gene expression are DNA methylation and histone post-translational modifications (especially histone deacetylation and methylation), as well as the production of noncoding RNAs. The epigenetic marks left by these modifications are catalyzed by different enzymes, which can act either as writers or erasers [4]. These enzymes work in coordination with another group of epigenetic players, called readers, which are proteins containing specialized domains that can identify and interpret an epigenetic mark in the chromatin structure and can recruit the right writer or eraser to its correct position [4]. One intriguing epigenetic reader is a ubiquitin-like containing plant homeodomain (PHD) and an interesting new gene (RING) finger domains 1 (UHRF1), which can act as a sensor of both types of epigenetic marks (DNA methylation and histone marks) and recruit the corresponding writers, DNA methyltransferase 1 (DNMT1) and G9A, or eraser histone deacetylase 1 (HDAC1), to the right place to catalyze the same epigenetic mark [5,6,7,8,9,10] (Figure 1).

**Figure 2:**
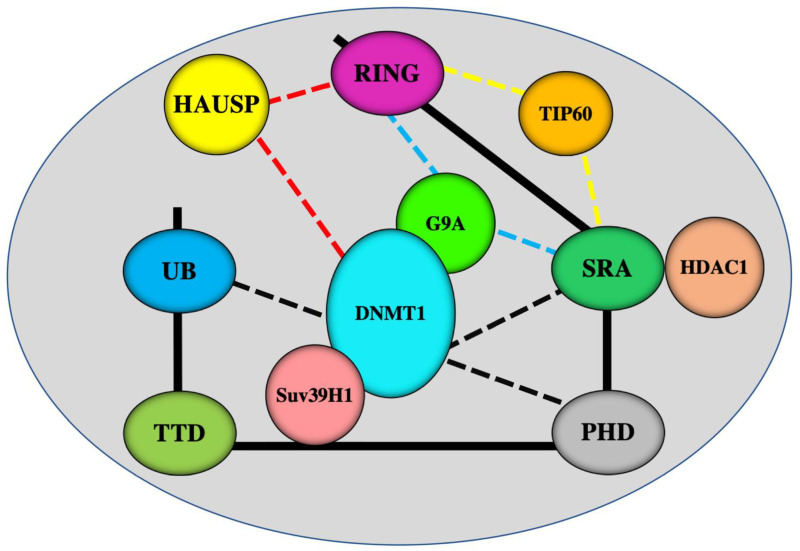
Schematic representation of interactions of UHRF1 domains with various epigenetic writers and erasers. UHRF1 uses its SRA domain to interact with DNMT1 [15,16,17] and HDAC1 [18]. UHRF1 can also interact with DNMT1 through PHD [36] and ubiquitin-like domain (UBL) [37,38] domains (black lines). HAUSP (herpes virus-associated ubiquitin-specific protease) interacts with both UHRF1 and DNMT1 [39,40] (red lines). Via its C-terminal region which covers the SRA and RING domains, UHRF1 interacts with histone methyltransferases G9a [21] (blue lines) and histone acetyltransferase Tip60 [19,20]. UHRF1 can interacts with another histone acetyltransferase Suv39H1 [41]. DNMT1 can also interact with Suv39H1 and G9a [42].

UHRF1 is an oncogene that is highly expressed in several blood malignancies and solid tumors [11,12,13,14]. It belongs to a large protein complex called the Epigenetic Code Replication Machinery “ECREM” (Figure 2) [12], which is formed through interactions between the different five domains of UHRF1 and several epigenetic writers and erasers (Figure 2) [11,12]. DNMT1, Tat Interacting Protein 60 (Tip60), a histone acetyltransferase, and the histone-lysine N-methyltransferases G9a and Suv39H1, are examples of the epigenetic writers, whereas HDAC1 and herpesvirus-associated ubiquitin-specific protease (HAUSP) serve as epigenetic erasers. The ability of UHRF1 to bind to DNMT1 [15,16,17], HDAC1 [18], Tip60 [19,20] and G9a [21], allows UHRF1 to serve as the master conductor for connecting DNA methylation to histone epigenetic markers (Figure 1) and consequently ensuring their inheritance through cell division [11,12,22]. Through these coordinated interactions, UHRF1 ensures a strong crosstalk between DNA methylation and histone post-transcriptional modifications (especially histone deacetylation and methylation), thereby silencing several TSGs, such as *p16^INK4A^*, *hMLH1* and *BRCA1,* throughout successive cell divisions, and facilitating the successful inheritance of the cancer phenotype by the daughter cells [5,11,12]. Interestingly, several studies have reported that either UHRF1 downregulation or targeting its functional domains can act as a trigger that reactivates several TSGs and enables cancer cells to undergo apoptosis, highlighting UHRF1 as a promising target for cancer drug development [23,24,25,26,27,28,29,30,31,32,33,34,35]. Inhibitors of UHRF1 activity and/or expression would conceivably prevent its ability to read the epigenetic markers, thereby also preventing its partners DNMT1, HDCA1 and G9a from acting out their roles as writers or erasers of epigenetic marks. The result of UHRF1 inhibition would, therefore, be the upregulation of the TSGs and subsequent activation of apoptosis pathway.

One interesting inhibitor of UHRF1 expression is thymoquinone (TQ), the most abundant biologically active component of black cumin seeds. Many in vitro and in vivo studies have shown that TQ exerts inhibitory effects on a number of different human cancers, including glioblastoma, breast carcinoma, leukemia, and lung, prostate, pancreatic, head and neck, cervical, and liver cancers [43,44,45,46,47,48,49]. TQ exerts its cytotoxic activities against tumor cells by several different mechanisms, including inhibition of cell division, promotion of cell cycle arrest, activation of ROS production, induction of apoptosis and inhibition of tumor angiogenesis and metastasis [50,51]. When compared to its effects on cancer cells, TQ has no or only mild cytotoxic effects on matched normal cells, such as normal human fibroblast cells [52], normal human gastric epithelial cells [53], primary normal neuronal cells [54], normal human astrocytes [55] and normal oral epithelial cells [56]. Although several in vitro and in vivo studies have demonstrated the therapeutic potential of TQ as an anticancer drug for both blood malignancies and solid tumors, there is a lack of clinical studies evaluating TQ in cancer patients. This might be attributed to the poor pharmacokinetics and chemical stability of TQ. Indeed, TQ is heat and light-sensitive, and it has poor solubility in aqueous media, which affects its biodistribution [57,58]. Additionally, covalent binding of TQ to serum albumin, and hepatic metabolism of TQ into hydroquinone, leads to significant loss of anticancer activity after oral administration [57]. To overcome these limitations, TQ could be loaded into nanoscale systems. In this context, TQ-loaded nanoparticles have demonstrated better anticancer activity than free TQ due to enhanced bioavailability and cellular uptake [59].

Of particular interest to this review is that several studies have now shown that TQ can target the epigenetic reader UHRF1 as well as its partners, the epigenetic writers (DNMT1 and G9A) and erasers like HDAC1 [23,60,61,62,63,64,65,66] (Table 1).

The TQ-induced inhibition of these epigenetic players is associated with an upregulation of several TSGs that are known to be repressed in several tumors through epigenetic mechanisms. Indeed, TQ can induce the degradation of UHRF1 through a fast autoubiquitination process involving the UHRF1 RING domain, which has a specific E3 ubiquitin ligase activity [23]. Interestingly, UHRF1 ubiquitination was not observed in TQ-treated cells that expressed a mutant form of UHRF1 with a specifically modified RING domain, indicating that the RING domain of UHRF1 undergoes autoubiquitination in response to TQ treatment [23]. Moreover, the deubiquitinase HAUSP, which is known to protect UHRF1 from degradation by the proteasome [39,40], was also downregulated in response to TQ, suggesting that TQ could be the trigger for the autoubiquitination of UHRF1 by an as yet unknown mechanism [23]. Nevertheless, it is absolutely established that the dissociation between HAUSP and UHRF1 is involved.

An effective epidrug for cancer therapy should consider the epigenetic code as a whole, rather than a single pharmacological target [67,68]. Single epitarget therapies suffer from some significant limitations, particularly the emergence of drug resistance and the triggering of adverse reactions [69]. Identifying compounds with multitargeting properties that are active against epigenetic marks should overcome these limitations. TQ, through its ability to target the expression of both the epigenetic reader UHRF1 and its preferred partners DNMT1, HDAC1 and G9a, is clearly a potential candidate as a multitarget epidrug with the capacity to reverse the epigenetic code of cancer cells as a whole, while allowing the reactivation of TSGs. This review highlights the increasing evidence for a role of TQ as a potential multitarget epidrug for the treatment of blood malignancies and solid tumors through its targeting of the UHRF1/DNMT1/HDAC1/G9a protein complex, in which UHRF1 functions as the hub protein. This review also deciphers the clues supporting a specific targeting of UHRF1 by TQ, with subsequent regulatory effects on the other UHRF1 partners.

## 2. Role of the DNMT1/HDAC1/G9a Complex in Epigenetic Silencing of TSGs

Inactivation of TSGs through epigenetic mechanisms (DNA methylation and histone posttranslational modifications) is one of key factors that promotes the onset of cancer. In cancer cells, the methylation profile is characterized by a global genome hypomethylation, accompanied by a hypermethylation of TSG promoters. The hypermethylation of the CpG islands in TSG promoters, catalyzed by DNMT1, is a significant event in the origin of many cancers [70,71]. Many TSGs, such as *RB1*, *VHL*, *p16^INK4a^*, *BRCA1*, *HIC-1*, *MLH1, RUNX3, RASSF1A, FOXO4, PPARG, STK4, PML* and *KISS1*, are silenced in tumors by hypermethylation of their promoters [1,11,12]. TSGs regulate several signaling pathways involved in cell proliferation, the cell cycle, DNA repair, invasion, apoptosis and angiogenesis, all of which are involved in the initiation and/or the development of cancer [11,13].

Apart from creating an imbalance in DNA methylation, aberrant histone post-translational modifications can also drive the epigenetic inhibition of TSGs in cancers. Aberrant histone post-translational modifications are particularly prevalent in cancer cells [72,73], with post-translational histone modifications usually occurring in the early stages of tumor development and accumulating during tumorigenesis [74,75]. The post-translational modifications occurring at certain sites on histones H3 and H4 are among the most important modifications that exert effects on gene expression [76,77,78]. This modification process is mediated by two types of enzymes with opposite activity: histone acetyltransferases (HATs) and histone deacetylases (HDACs).

The downregulation or upregulation of HATs is accompanied by tumorigenesis or poor prognosis [79,80]. The Tip60 histone acetyltransferase can acetylate histone proteins, such as histone 2A on lysine 5 (H2AK5) [81], as well as several nonhistone proteins, including p53 and Myc [82], and Tip60 can serve either as an oncogene or as a tumor suppressor [83]. Several tumors, such as skin cancer [84] and osteosarcoma [85], show overexpressed Tip60, whereas other tumors, including lung [86] and breast cancer [87], show low tumor expression levels of Tip60.

The HDACs, a family of four enzyme subclasses, also have a vital role in tumorigenesis, and are attracting attention due to their contributions to several biological processes, as well as their interactions with other epigenetic enzymes [79]. Changes in HDACs expression in tumors usually result in aberrant deacetylation, leading to activation of TSGs. HDAC1, a class I member, is considered an important epigenetic player mediating histone deacetylation [88,89,90]. HDAC1 is highly expressed in many human tumors, and its overexpression is associated with poor outcomes and tumor progression, thereby identifying this epigenetic eraser as a promising target for cancer therapy [88,91,92,93]. Downregulation of HDAC1 inhibits cell proliferation and cell cycle progression and induces apoptosis in many human tumors, including breast and colon cancer cells, ovarian cancer and lung cancer [90,94,95]. Clinically, HDAC1 overexpression at the mRNA and proteins levels is associated with the clinical features and poor prognosis of patients with breast [96], lung [88] and gastric cancer [97], supporting the idea that the inhibition of HDAC1 activity and/or HDAC1 expression could be a potent strategy for cancer therapy.

Epigenetic changes involving histone methylation/demethylation are dynamically regulated by two families of enzymes: histone lysine methyltransferases (KMTs) and lysine-specific histone demethylase (KDMs) [98]. Lysine residues can be mono, di or trimethylated, and this process is regulated by the expression levels and the recruitment of KMTs/KDMs to chromatin. The methylation of lysine within histone tails, catalyzed by KMTs, plays a central role in the control of gene transcription [99,100]. G9a, also known as EHMT2 (euchromatic histone-lysine N-methyltransferase 2) and KMT1C (lysine methyltransferase 1C), is one of the major euchromatic methyltransferases [101,102]. The di- or trimethylation of lysine 9 of histone 3 (H3K9me2 or H3K9me3) mediated by the KMT G9a is considered one of histone changes with a known role in gene silencing, whereas the methylation of lysine 4 (H3K4me) on the same histone is related to gene activation [103,104]. Several studies have shown that G9a is overexpressed in a panel of human cancers, and that its high expression levels are associated with unfavorable clinicopathological parameters and poor survival [105,106,107,108]. Interestingly, the depletion of G9a is sufficient to induce a reactivation of TSGs and inhibition of cancer cell proliferation [109,110,111].

Several other studies have also reported a coordinated activity between several epigenetic factors, including DNMT1, HDAC1, G91 and Suv39H1, during DNA synthesis that maintains the transmission of the epigenetic code [42,112,113,114,115,116]. In this context, DNMT1 was shown to physically interact with both the H3K9 histone methyltransferases G9a and Suv39H1 [42]. DNMT1 colocalized with G9a at replication foci during DNA replication, while DNMT1 colocalized with Suv39H1 on heterochromatic regions predominantly before cell division [42]. The DNMT1/HDAC1/Suv39H1 complex, in coordination with other factors, was found to regulate the expression of the estrogen receptor-a (ER) in breast cancer cells [116].

A key remaining question is whether a principal conductor exists that coordinates all these epigenetic factors to orchestrate the precise timing of the recruitment of the correct enzyme to its right place. In this review, we propose that UHRF1 is a likely candidate. Based on its structure and its multiple interactions with several writers and erasers, UHRF1 can direct coordinated crosstalk between DNA methylation and histone posttranslational modifications, thereby making it a probable candidate as the leader in the ECREM complex.

## 3. A master Role for UHRF1 in the ECREM Complex Driving Epigenetic Inhibition of TSGs

In tumors, no clear mechanisms are yet identified that explain the maintenance of the inheritance of a silenced TSG from a mother cancer cell to the daughter cells during cell division. However, the functioning of UHRF1 as the conductor and hub protein in its complex could ensure this transmission during cell division [5,6,12,14,15,16,20,22,36,78]. Indeed, several in vitro and in vivo studies have reported the detection of UHRF1 overexpression in many human cancers, and that this overexpression is a crucial factor in the epigenetic silencing of various TSGs and leads to enhanced cell proliferation, cell cycle progression, and suppression of apoptosis [11,12]. UHRF1 uses its different domain functions to repress the expression of TSGs through several mechanisms that involve TSG promoter hypermethylation via the physical interaction with DNMT1, HDAC1-mediated histone deacetylation, and G9a-catalyzed histone 3 methylation [11,12] (Figure 1).

The induction of TSGs silencing is well documented to occur by hypermethylation of CpG islands located within TSGs promoters. However, the mechanisms by which the CpG islands are specifically targeted is still unclear. One hypothesis is that the hypermethylation of the CpG islands in the TSG promoters is driven by a mechanism involving a protein that can bind to DNA and guide DNMT1 to its correct place at the right time during DNA replication. UHRF1 is a likely candidate because of its high affinity for hemimethylated vs. nonmethylated DNA [117], and its direct interaction with DNMT1 [15,16,17,36]. These capabilities give UHRF1 the necessary duality to allow the successful transfer of DNA methylation patterns, including the hypermethylation of TSGs. During DNA replication, the SRA domain of UHRF1 can recognize methylated CpG sites (hemimethylated DNA) by flipping out the methylated cytosine. In addition, via the same domain, UHRF1 can recruit DNMT1 and guide it to methylate the unmethylated cytosine of the newly synthetized DNA strand [117] (Figure 1). UHRF1 can also interact with DNMT1 through its PHD domain [36] and UBL domain [37,38] (Figure 2). Beside the role of SRA domain of UHRF1 in the binding to hemimethylated DNA and the recruitment of DNMT1 to sites of methylation DNA [117], UHRF1 has a well-established role through its RING domain in the ubiquitylation of H3 and DNMT1, targeting for sites of hemimethylated DNA [118,119]. Indeed, UHRF1 uses the ubiquitin ligase activity of its RING domain to ubiquitinate H3. The reading and writing this epigenetic mark (H3 ubiquitination) by UHRF1 is a prerequisite for the binding of DNMT1 to ubiquitylated histone H3 [120] to ensure a faithful recruitment of DNMT1 to sites of hemimethylated DNA [22,118,119]. Moreover, the recruitment of DNMT1 to DNA methylation sites is also regulated by H3 deubiquitylation through a mechanism involves HAUSP, another member of ECREM complex [121]. HAUSP was shown to interact with DNMT1 and is recruited to sites of DNA methylation during DNA replication, and this recruitment requires UHRF1 [121]. HAUSP induced the deubiquitylation of ubiquitylated histone H3 in vitro, while HAUSP depletion in cancer cells resulted in enhanced histone H3 ubiquitylation [121]. This suggests that HAUSP has a key role in the regulation of maintenance of DNA methylation through UHRF1-dependent deubiquitylation of ubiquitylated histone H3. However, HAUSP and DNMT1 appear to behave as independent proteins at replication foci, since global DNA methylation levels were not notably altered in cells with HAUSP knockout [122,123].

Several works have shown that both histone methylation and acetylation work together with DNA methylation to exert inhibitory effects on the expression of TSGs in cancer cells through mechanisms that remain incompletely understood [124,125,126,127,128]. Through its SRA domain, UHRF1 can directly bind to HDAC1 and recruit it to methylated promoter regions of the TSGs *p16^INK4A^* and *p14^ARF^*, resulting in their silencing by a histone deacetylation process [18]. In the same context, renal cell carcinoma (RCC) tumors show high expression levels of UHRF1 compared to normal renal tissues, and this overexpression is associated with a decreased expression of the tumor suppressor gene *TXNIP* [129]. UHRF1 was shown to recruit HDAC1 to the *TXNIP* gene promoter and mediate the deacetylation of histone H3 on the lysine 9 (H3K9), resulting in an epigenetic inhibition of TXNIP expression [129]. Interestingly, UHRF1 downregulation in RCC cell lines induced the upregulation of TXNIP expression and apoptosis, suggesting that UHRF1 inhibits the expression of TXNIP in RCC through epigenetic mechanisms, thereby promoting tumor progression [129].

The UHRF1/HAUSP/DNMT1 complex was also detected on the promoters of *HHIP* and *IGFBP3*, two key TSGs in hepatoblastoma, and this interaction caused the inhibition of these genes [130]. Silencing of *HHIP* and *IGFBP3* genes was associated with an increase in the dimethylation of histone 3 on lysine 9 (H3K9me2) [130], which is a well-documented repression mark in cancer [131,132]. Interestingly, the depletion of UHRF1, but not of its partner HAUSP, significantly increased the expression of the *HHIP* and *IGFBP3* genes and decreased the H3K9me2 mark at the *HHIP* and *IGFBP3* TSG loci, leading to the inhibition of hepatoblastoma cell growth [130]. Similarly, UHRF1 was also shown to recruit DNMT1 to the promoter of the tumor suppressor gene *BRCA1* leading to *BRCA1* inhibition through methylation of its promoter [133]. Besides guiding DNMT1, UHRF1 also recruited HDAC1 and G9a to the *BRCA1* loci, resulting in histone 3 deacetylation and methylation, respectively, and facilitating the silencing of *BRCA1* [133].

Taken together, the findings of these studies support the idea that UHRF1 overexpression is one of the primary causes of cancer pathogenesis, and that UHRF1 exerts direct inhibitory effects on various TSGs through a coordinated recruitment of several epigenetic players, namely DNMT1, HDAC1 and G9a, to their correct places on the chromatin to catalyze the right epigenetic mark (Figure 1). These studies also reinforce the view that DNMT1, HDAC1 and G91 might coregulate the expression of TSGs in cancer, and that this process is directly under the control of the epigenetic reader UHRF1 (Figure 1). Thus, understanding the role of the UHRF1/DNMT1/HDAC1/G9a complex in reading the epigenetic marks (DNA methylation and histone marks) in cancer will allow the development of a new generation of multitarget epidrugs. These drug candidates may have value in targeting UHRF1 as the principal conductor, with subsequent regulatory effects on the other partners, DNMT1, HDAC1 and G91.

## 4. UHRF1 Is a Main Target of Natural Compounds Exhibiting Anticancer Properties

UHRF1 has been reported to be a target of several natural compounds or derivatives that exhibit anticancer properties by downregulating UHRF1. These compounds include TQ [11,12,23,60,61,62,63], curcumin [134], epigallocatechin-3-gallate [24,135], anisomycin [136], dihydroartemisinin [40], emodin [137], hinokitiol [33], shikonin [138], and luteolin [139,140], which can all upregulate many TSGs. Of these natural products, epigallocatechin-3-gallate and TQ have been shown to specifically target the SRA and RING domains of UHRF1, respectively. Our previous work revealed that epigallocatechin-3-gallate (EGCG) induced cell cycle arrest and apoptosis in Jurkat cells by the downregulation of UHRF1 and DNMT1, and the upregulation of the tumor suppressor *p16^INK4A^* [24]. A significant decrease in UHRF1 binding to the *p16^INK4A^* promoter was detected in response to EGCG but not in nontreated Jurkat cells. Overexpression of wild-type UHRF1 decreased the p16^INK4A^ protein expression in the presence of EGCG, but the overexpression of UHRF1 mutations that specifically targeted two regions of the UNRF1 SRA domain did not decrease p16^INK4A^ expression, indicating that UHRF1 requires a functional SRA domain to bind DNA and recruit DNMT1 to chromatin [24]. We also found that TQ induces auto-ubiquitination of UHRF1 and subsequent degradation in cancer cells [23] by targeting its RING domain, which is the only domain of the UHRF1 structure that exhibits enzymatic activity [5,141].

## 5. The UHRF1 Protein Complex Is a Main Target of TQ

### 5.1. Inhibitory Effects of TQ on UHRF1

**Figure 3:**
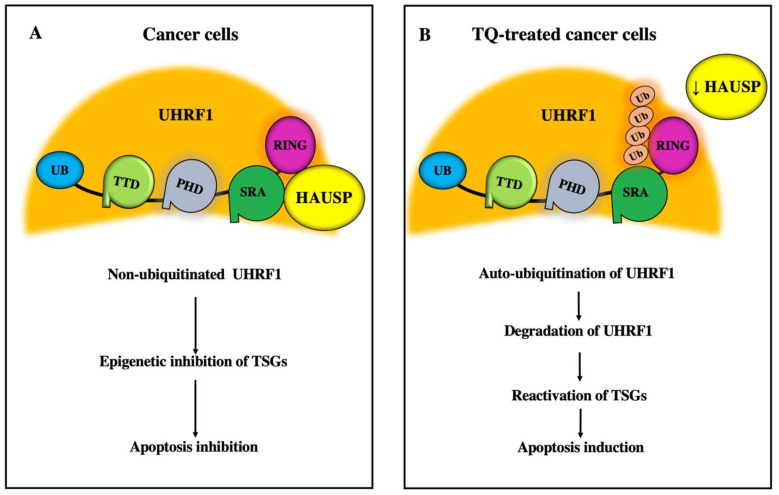
Schematic representation of TQ-induced HAUSP/UHRF1 deregulation and the related events. (**A**). In cancer cells, UHRF1 is protected from degradation through a direct interaction with HAUSP leading to epigenetic inhibition of TSGs which subsequently inhibits apoptosis. (**B**). Exposure of cancer cells to TQ induces a decrease in the expression of HAUSP which allows an autoubiquitination of UHRF1 through its E3 ubiquitin ligase activity as a first step for its degradation later inducing the reactivation of TSGs and apoptosis.

TQ decreases the expression of the UHRF1 protein in p53-deficient Jurkat cells in parallel with an upregulation of the tumor suppressor p73 [63]. The depletion of p73 in Jurkat cells can protect UHRF1 from TQ-induced degradation, indicating that the high expression levels of UHRF1 detected in either blood cancers or solid tumors with p53 mutations [142,143,144,145] could be attributed to a loss of p73 expression. Similarly, the low expression levels of UHRF1 were also restored in TQ-treated Jurkat cells when phosphodiesterase 1A (PDE1A) was overexpressed, while p73 expression was significantly repressed, suggesting that the TQ-induced downregulation of UHRF1 can be attributed to the inhibitory effects of TQ on PDE1A through some as yet unknown mechanism [62]. Recently, our team demonstrated that TQ induces the degradation of UHRF1 through a rapid auto-ubiquitination process involving its RING domain, and that this mechanism appeared to be correlated with a decrease in the expression of its partner HAUSP [23]. Interestingly, no UHRF1 ubiquitination was detected in the TQ-treated cells that expressed a UHRF1 mutation that specifically targeted its RING domain, again supporting an autoubiquitination of UHRF1 through its RING domain in response to TQ [23]. These findings indicate that UHRF1 is protected from degradation in cancer cells through its direct interaction with HAUSP, leaving it free to inhibit TSGs, with the subsequent inhibition of apoptosis (Figure 3A). Conversely, TQ treatment decreased the expression of the HAUSP protein, removing the protection of UHRF1 and leaving it vulnerable to autoubiquitination through its E3 ubiquitin ligase activity, with subsequent induction of apoptosis (Figure 3B). Taken together, these study findings suggest that UHRF1 is a main target of TQ, which triggers autoubiquitination of UHRF1 through its RING domain, thereby allowing the reactivation of several TSGs, with subsequent suppression of cell proliferation, promotion of cell cycle arrest, and induction of apoptosis. This also suggest that TQ could be a promising epidrug that acts via a specific inhibition of UHRF1 expression levels in cancer cells without affecting its expression in normal human cells.

In human astrocytoma cells and Jurkat cells, TQ was shown to induce a concentration- and time-dependent degradation of UHRF1 and α/β tubulin, while no similar effect was observed in normal human fibroblast cells, again suggesting that TQ exerts a selective effect on UHRF1 [52]. At present, no mechanism can explain why TQ selectively induces UHRF1 degradation in cancer cells without affecting its expression levels in normal cells. The UHRF1 protein is essential for cell proliferation [17,146,147] and is overexpressed in rapidly multiplying cancer cells; therefore, one possibility is that normally growing cells are less sensitive to TQ than their cancer cell counterparts that have high expression of UHRF1. This hypothesis is supported by several in vivo and in vitro studies that have reported a greater sensitivity of cancer cells than normal cells to the inhibitory effects of TQ [148,149,150,151].

### 5.2. Inhibitory Effects of TQ on DNMT1 Expression and Activity

**Figure 4:**
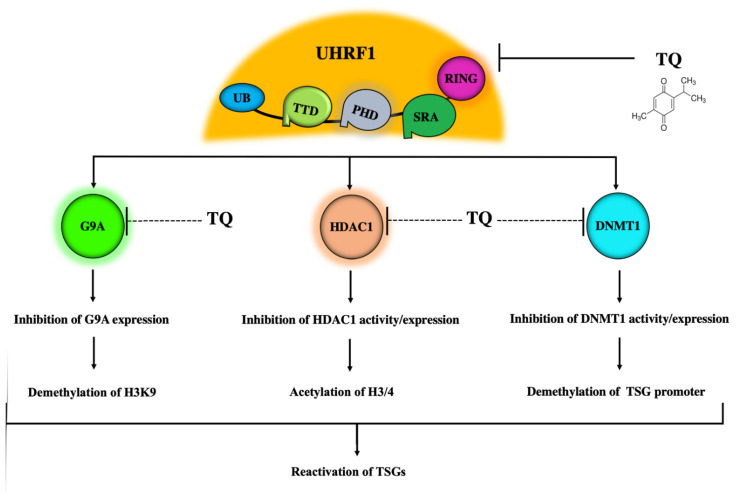
Schematic representation of TQ as a multitargeting single epidrug. TQ can directly inhibit the expression of UHRF1 protein leading to the inhibition of activity and/or activity of its partners DNMT1, HDAC1 and G9A. TQ could also target DNMT1, HDAC1 and G9A in UHRF1-indpendent mechanisms. DNMT1 inhibition leads to demethylation of TSG promoter. HDAC1 inhibition enhances the acetylation of histones 3 and 4. G9A inhibition causes demethylation of H3K9. All these TQ-induced effects lead to the reactivation of TSGs.

In cancer cells, promoters of TSGs are hypermethylated by DNMT enzymes, leading to the inhibition of TSG expression and subsequent defects in apoptosis. Decitabine and azacytidine are the two DNMT inhibitors approved for the therapy of blood tumors, such as myelodysplastic syndrome and acute myeloid leukemia [152]. Several hematological adverse effects, as well as development of resistance, have been reported following the treatment with these drugs, so a persistent demand exists for new DNMT inhibitors with low toxicity for the treatment of blood cancers, as well as solid tumors. TQ shows promising inhibitory effects on several cancers through its targeting of several mechanisms, including the upregulation of TSGs, and it shows only mild cytotoxic effects on matched normal cells, making it a promising hypomethylating agent through its specific inhibition of DNMT1. Thus, due to its significantly lower cytotoxicity to normal human cells, TQ could achieve similar or better clinical outcomes compared with the approved DNMT inhibitors. Several in vitro and in vivo studies support its potential clinical application, as TQ can induce the expression of several TSGs, including *PTEN* [153], BRCA1 [154], *HIC1* [154], *p73* [155] and *p16*^INK4a^, which are known to be epigenetically silenced in various tumors [148]. A recent study has shown that TQ exerts in vitro cytotoxicity effects against leukemia by inhibiting the activity of DNMT1 and inducing global DNA hypomethylation [64]. A molecular docking study suggests that TQ interacts with the catalytic pocket of DNMT1, but the exact binding model of TQ to DNMT1 remains unknown and needs further investigation [64]. TQ was also able to inhibit the DNMT1 methylation activity in a dose-dependent manner. Treatment of ML-1, Kasumi-1 and MV4-11 acute myeloid leukemia cells with TQ induced a significant decrease in the expression of DNMT1 and DNMT3A proteins. Mechanistically, TQ decreased the expression of DNMT1 through the disruption of the Sp1/NFkB complex from its promoter and decreased the expression of the DNMT3A protein through the upregulation of miR-29b, which directly binds to the 3′-UTR of DNMT3A [156]. Interestingly, in blasts of leukemia patients, TQ decreased the expression of DNMT1 and DNMT3A at both the mRNA and protein levels and induced apoptosis [64]. These findings indicate that the anticancer actions of TQ involve the inhibition of both the activity and expression of DNMT1 for reactivation of TSGs and suggest that TQ is an efficient epigenetic drug for leukemia therapy. In support of this idea, TQ was shown to decrease the expression of DNMT1 in parallel with an upregulation of the tumor suppressor gene *p73* [63], which is known to be repressed in blood tumors by hypermethylation of its promoter [155]. Indeed, the p73 promoter was methylated in bone marrow samples from adult patients with myelodysplastic syndromes, and its hypermethylation was associated with poor prognosis [155]. Interestingly, treatment of bone marrow samples with the antileukemic drug cytarabine was able to restore the expression of p73 and increased its levels to promote the induction of apoptosis [155]. In the same context, TQ increased the expression levels of *p16*^INK4a^, a downstream target of p73 and epigenetically silenced several tumors through several mechanisms, including the hypermethylation of its promoter [63,148,157,158,159,160]. Similarly, the TQ-induced apoptosis in triple-negative breast cancer cells was correlated with an increased expression of two TSGs, *BRCA1* and HIC1 [154], which also show well-documented repression in various tumors through hypermethylation of their promoters [161,162,163,164,165,166]. In human breast cancer cells, TQ can increase mRNA expression of the tumor suppressor *PTEN* [153], known to be epigenetically inhibited in various cancers [167,168,169]. Recently, TQ was shown to increase the expression of a panel of TSGs, including *DLC1, SALL4, PPARG, DDIT3, FOXO6, CYP1B1, TET2* and *ST7* in Jurkat cells [60]. The TQ-induced upregulation of these TSGs was associated with a significant decrease in the expression of several DNA methyltransferases, notably DNMT1, DNMT3Aand DNMT3B, and the induction of apoptosis [60]. Taken together, these studies suggest that TQ can inhibit the activity and/or expression of DNMT1 through several mechanisms to cause the demethylation of TSGs promoters, with the subsequent reactivation of the relevant TSGs and the induction of apoptosis. The interaction of the SRA domain of UHRF1 with DNMT1, together with the inhibition of expression of the UHRF1 protein by TQ, raises the possibility that TQ directly inhibits DNMT1 and/or indirectly operates through a mechanism that involves the inhibition of UHRF1 expression (Figure 4).

### 5.3. Inhibitory Effects of TQ on HDAC1

In addition to the silencing of their promoters by hypermethylation, TSGs can be silenced by histone hypoacetylation at target TSG loci through increased HDAC or decreased HAT activity. Vorinostat and panobinostat, two clinically approved inhibitors of HDACs, have shown promising clinical benefits for patients with lymphoid and myeloid malignancies [170,171,172,173]. TQ-induced upregulation of TSGs in cancer could be attributed in large part to the inhibitory effects of TQ on the UHRF1/DNMT1/HDAC complex, with subsequent apoptosis induction. In this context, TQ-induced upregulation of *p16^INK4A^* and apoptosis was associated with a decrease in the expression of UHRF1, DNMT1, and HDAC1 proteins [63]. This suggests that upregulation of *p16^INK4A^* results from demethylation of its promoter, as well as to an acetylation process due to a reduction in HDAC1 binding to regions located at *p16^INK4A^* promoter. In line with this possibility, the UHRF1/HDAC1 complex was located at methylated promoter regions of *p16^INK4A^* and this led silencing of *p16^INK4A^* through histone deacetylation [18]. TQ treatment induced cell proliferation, cell cycle arrest and apoptosis in the AsPC-1 and MiaPaCa‑2 human pancreatic ductal adenocarcinoma cell lines through the upregulation of the proapoptotic genes *p53* and *BAX,* and the downregulation of the antiapoptotic gene *BCL2* [65]. TQ-induced apoptosis was associated with a significant reduction in HDAC activity and a decrease in the expression of HDACs 1, 2, and 3 at the mRNA level [65]. Additionally, TQ reduced the tumor size in human pancreatic ductal adenocarcinoma xenografts, and this effect was also associated with a significant decrease in the expression of HDACs 1, 2, and 3 [65]. Interestingly, the TQ-induced inhibition of HDAC activity and expression was associated with an increase in the acetylation of histone 4 at lysine 12 (H4 Ac-K12) [65], indicating that TQ can increase the expression of TSGs in cancer by decreasing the activity and expression of HDACs, and by increasing histone acetylation. Similar findings were reported in breast cancer cells, where TQ was shown to interact with human HDACs and to inhibit in vitro the global HDAC activity [66].

Exposure of MCF-7 breast cancer cells to TQ inhibited HDAC activity and increased the expression of two TSGs, *p21* and *Maspin* [66]. In the same context, TQ-induced upregulation of various TSGs in Jurkat cells was associated with a decrease in the expression of HDACs 1, 4 and 9 [60]. TQ also significantly decreased the mRNA expression of HDAC1 in Jurkat cells, as well as in a human breast cancer cell line (MDA-MB-468 cells) [60]. Several in vitro and in vivo studies have shown that TQ exerts synergistic inhibitory effects when combined with several other clinically approved drugs, such as tamoxifen [174,175], docetaxel [176,177], cisplatin [178] and 5-fluorouracil [49,179], by targeting several signaling pathways. Recently, a combination of TQ and the anticancer agent difluoromethylornithine (DFMO) showed a significant synergistic induction of apoptosis in Jurkat cells [61]. Interestingly, this induction was associated with a dramatic decrease in the mRNA expression of UHRF1, DNMT1 and HDAC1 [61].

These findings indicate that TQ could act as an HDAC inhibitor, changing the epigenetic state of histones through the inhibition of histone deacetylation and an induction of histone acetylation, thereby triggering apoptosis via the upregulation of TSGs. This mechanism could be mediated by the inhibition of UHRF1 by TQ. Since UHRF1 physically interacts with HDAC1, and UHRF1 is also targeted by TQ, this also suggests that the TQ-induced HDAC1 inhibition is due to the inhibitory effects of TQ on UHRF1 (Figure 4).

### 5.4. Inhibitory Effects of TQ on G9A

The histone lysine methyltransferase G9a is overexpressed in many tumors and is a well‑characterized drug target for cancer treatment [180]. G9a methylates H3K9me2 or H3K9me3, which are repressive epigenetic modifications, and leads to transcriptional silencing of target TSGs in cancers. In vitro and in vivo evaluations of small-molecule inhibitors of G9a have shown anticancer effects in both hematologic and solid tumors [181,182]. TQ treatment, which increases the expression of BRCA1 in triple-negative breast cancer cells through an unknown mechanism [154], was also recently found to induce a significant decrease in the expression of G9a in Jurkat cells and in breast cancer cells, in parallel with the upregulation of several TSGs [60]. The known interaction between UHRF1 and G9a, both in vitro and in vivo [21], is also implicated in the epigenetic silencing of BRCA1 in sporadic breast cancer [133]. Therefore, TQ could quite possibly inhibit G9a and/or delocalize it from chromatin through its effects on UHRF1. This would result in the demethylation of H3K9 and a subsequent upregulation of TSGs, including BRCA1 (Figure 3).

## 6. Conclusions

The increasing role of the UHRF1/DNMT1/HDAC1/G9a complex in the epigenetic silencing of many TSGs in cancer supports the targeting of this multiprotein complex as a valuable approach for developing multitarget single epidrugs. UHRF1 contains specialized domains that render it an epigenetic reader of both hemimethylated DNA and histone marks and allow it to recruit the right enzyme (DNMT1, HDAC1 or G9a) to the right place with precise timing during cell division, making it a promising target for epigenetic therapy. Inhibiting UHRF1 reverses the cancer cell epigenetic code as a whole (DNA methylation and the histone code) and leads to reactivation of TSGs. Today, several epigenetic drugs that target DNMT1 and HDAC1 are already approved for clinical uses. TQ, by virtue of its ability to induce autoubiquitination of UHRF1 through its RING domain followed by UHRF1 degradation, could also be added to this arsenal. The most interesting aspect of the mode of action of TQ is that a single drug has the ability to down-regulate several members of a macromolecular complex and this probably contributes to its high efficiency as an anticancer drug. This also shed lights on the possibility that TQ acts on upstream regulatory pathways common to all the ECREM members or at least to UHRF1, DNMT1, G9a and HDAC1.

## Figures and Tables

**Table 1:** Epigenetic targets of thymoquinone in cancer.

Epi-Target	Role of Epi-Target	Experimental Model	Mechanisms of Action	References
UHRF1	Reader	Human cervical carcinoma HeLa cells. T-ALL	TQ targeted the E3 ubiquitin ligase activity of UHRF1 resulting in an auto-ubiquitination of UHRF1 likely through the downregulation of HAUSP	[23]
		T-ALL	TQ upregulated p73 expression and cleaved caspase 3 leading to UHRF1 degradation	[63]
		T-ALL	TQ decreased the expression of PDE1A leading to the upregulation of p73 and downregulation of UHRF1	[62]
		T-ALL Human breast cancer cells	TQ decreased the expression of mRNA UHRF1 in dose-dependent mechanism	[60]
DNMT1 DNMT3A DNMT3B	Writer	Human acute myeloid leukemia cells Patient primary cells	TQ inhibited DNMT1 activity and decreased its expression through the disruption of Sp1/NFkB complex from DNMT1 promoter. TQ decreased the expression of DNMT3A through the upregulation of miR-29b, known to directly bind to the 3′-UTR of DNMT3A	[64]
		T-ALL	TQ decreased the expression of DNMT1 protein	[63]
		T-ALL	TQ decreased the expression of DNMT1, 3A,3B	[60]
HDAC1 HDAC2 HDAC3 HDAC4 HDAC9	Eraser	T-ALL	TQ decreased the expression of HDAC1 protein	[63]
		T-ALL	TQ decreased in the expression of HDAC1, 4 and 9	[60]
		T-ALL Human breast cancer cells	TQ decreased the expression of mRNA HDAC1 in dose-dependent mechanism	[60]
		Human pancreatic ductal adenocarcinoma cells. Human pancreatic ductal adenocarcinoma xenografts.	TQ inhibited HDAC activity, decreased the expression of HDAC 1, 2, 3 at mRNA levels and increased the acetylation of histone 4 at lysine 12 (H4 Ac-K12)	[65]
G9A	Writer	T-ALL Human breast cancer cells	TQ decreased the expression of mRNA G9A in dose-dependent mechanism	[60]

## Data Availability

No new data were created or analyzed in this study. Data sharing is not applicable to this article.

## Abbreviations

DNMT1	DNA methyltransferase 1
ECREM	Epigenetic Code Replication Machinery
EGCG	Epigallocatechin-3-gallate
HAUSP	Herpes virus-Associated Ubiquitin-Specific Protease
HDAC1	Histone deacetylase 1
PDE	Phosphodiesterase
PHD	Plant Homeo Domain
RING	Really Interesting New Gene domain
SRA	Set and Ring Associated domain
TSG	Tumor suppressor gene
TQ	Thymoquinone
TTD	Tandem Tudor Domain
UBL	Ubiquitin-like domain
UHRF1	Ubiquitin-like with PHD and RING Finger domains 1
